# Supplementary material for: Missed Opportunities for Vaccination and Associated Factors among Children Attending Primary Health Care Facilities in Cape Town, South Africa: A Pre-Intervention Multilevel Analysis
Source: Vaccines (Basel). 2022 May 16;10(5):785. doi: 10.3390/vaccines10050785 (PMC9144782; doi:10.3390/vaccines10050785)
Supplement: Supplementary file 1 [file vaccines-10-00785-s001.zip › vaccines-1679142-supplementary.pdf]

**MISSED OPPORTUNITIES FOR VACCINATION (MOV) AMONG CHILDREN IN CAPE TOWN**

**QUESTIONNAIRE:**

Respondent ID___________________

Date of interview ________________

Name of interviewer______________________

**HEALTH FACILITY CHARACTERISTICS**

1. Name of health facility__________________________________
2. Name of sub-district _________________________________
3. Type of health facility 1] Comprehensive health centre 2] Clinic
4. Facility ownership 1] Provincial 2] City of Cape Town 3] Others (Specify) _________________________
5. Total number of health workers _________________________
6. Average number of patients seen _______________________________
7. Is there a designated vaccinator primarily stationed in vaccination clinic? 1] Yes 2] No
8. Does the facility have designated immunization days 1] Yes 2] No
9. Has there been vaccine cold-chain and storage challenge in this facility in the past 3 months, such as prolonged electricity outage, bad refrigerators, lack of cold boxes etc? 1] Yes 2] No
10. Any vaccine stock-out in the past 3 months? 1] Yes 2] No

**SECTION A: CHILD DATA**

1. Date of birth ___________________________
2. Age (in months) ___________________________________
3. Sex 1] Male 2] Female
4. Birth order 1] First 2] Second 3] Third 4] Others (specify)
5. Birth weight (in Kg) ___________________________________
6. Why did you bring the child to this health facility today? (Do not read out options) 1] For medical consultation (child is sick) 2] For vaccination 3] Growth and Development Check-up 4] Child is only accompanying (not for treatment/vaccination) 5] Hospitalization (Child was admitted or still on admission) 6] Others (Please specify)

**SECTION B: PARENT/CAREGIVER DATA**

7. Age ______________________________
8. Sex 1] Male 2] Female
9. Marital status 1] Single 2] Married 3] Separated 4] Divorced
10. What is your relationship with the child? 1] Mother 2] Father 3] Grandparent 4] Uncle 5] Aunt 6] Brother 7] Sister 8] Mother in law 9] Others (please specify)
11. Did mother attend antenatal care during index child's pregnancy? (If father or other caregivers, do you know if mother attended antenatal care during index child's pregnancy 1] Yes 2] No
12. Level of formal education 1] No formal education 2] Did not complete primary education 3] Completed primary education only 4] Completed secondary education only 5] Post-secondary education
13. Are you currently employed? 1] Yes 2] No
14. If employed, what is your monthly income 1] Less than R10 000 2]R10 000 to R30 000 3] Above R30 0000
15. By what means of transportation do you usually use to come to this facility? 1] Walk 2] Own transport 3] Public transport 4] Others (please specify)
16. How long does it take you to get to this health facility from your home? _______Hours ________ Minutes
17. What is the estimate cost of transportation to this place? ________________
18. Have you heard, seen any message on vaccination in the last 3 months? 1] Yes 2] No
19. If yes, where? 1] Radio 2] Television 3] Newspaper 4] Health facility 5] Telephone message 6] Facebook or Internet 7] Children's school 9] Mosque 10] Church 11] During home visit by health workers 12] Community meetings 13] Others (Please specify)
20. Do you feel you know the vaccines your child needs? 1] Yes 2] No 3] Not sure
21. If yes, do you feel that you know when the vaccines should be given? 1] Yes 2] No 3] Not sure
22. Has this child ever been vaccinated? 1] Yes 2] No
23. If no, why not? 1] The necessary vaccines or supplies were not available 2] I am not in favor of vaccination 3] My husband/the decision maker is not in favour of

vaccination 4] I have not visited the health facility on a vaccination day 5] I did not know that the child was eligible to be vaccinated 6] Others (Please specify)

24. Have you ever requested vaccination services for this child and child didn't get vaccinated? 1] Yes 2] No

25. If yes, why was child not vaccinated? 1] The health worker said it couldn't be done because the child was sick 2] There were no vaccines or there were no syringes or some other supply needed for vaccination 3] it was not a vaccination day 4] The vaccination area was closed 5] The person in charge of vaccination was not there 6] There would have been a long wait 7] We didn't have the Road to Health booklet (RtHB) with us 8] The hours for vaccination are limited 9] Others (Please specify)

26. In your home who makes the decision to vaccinate? 1] Father 2] Mother 3] Other relatives 4] Consensus of father and mother 5] Others (Specify) _____________

**SECTION C: USE OF ROAD TO HEALTH BOOKLET (RtHB) AND INFORMATION ON VACCINES ADMINISTERED**

27. Does your child have a Road to Health booklet (RtHB)? 1] Yes, and I have it with me 2] Yes, but I do not have it with me 2] No

28. If no, why don't you have a Road to Health booklet (RtHB)? 1] I lost it 2] I have never been given one 3] I don't know 4] Others (Please specify)

29. Why do you not have the Road to Health booklet (RtHB) or temporary vaccination documents with you today? 1] It is at the school/day care center 2] I left it at home because I forgot to bring it 3] I left it at home because I didn't know it was important to bring along 4] I lost it 5] The care has been damaged 6] I have never been given one 7] Because vaccination was not the reason for this visit 8] Others (Please specify)

30. What vaccines has the child received to date? (Obtain information only from Road to Health booklet (RtHB), vaccination register, or other temporary vaccination documents)

| Name of Vaccine (Antigen)/Dose | Date of administration |
|---|---|
| | |
| | |
| | |
| | |

| | |
|---|---|
| | |
| | |
| | |

31. Based on the above information and the EPI schedule (refer to the SA EPI schedule), are there vaccines/doses the child is eligible for by age that the child is yet to receive? 1] Yes 2] No

32. Have you ever lost the Road to Health booklet (RtHB)? 1] Yes 2] No

33. If yes, did you encounter difficulty getting it replaced? 1] Yes 2] No

34. Could you tell me what purpose the Road to Health booklet (RtHB) serves? 1] Don't know 2] To know what vaccines the child has had and which are missing 3] Overall health record and growth monitoring 4] Record and remind for return visit dates 5] Birth certificate and/or identification 6] Others (Specify)

**SECTION D: TODAY'S VISIT**

35. What day of the week is today? 1] Week day 2] Weekend (Saturday or Sunday)

36. Time of the visit 1] Morning 8:01 am – 12 noon 2] Afternoon (12:01pm to 5:59pm) 3] After hours (6:00pm – 8:00am)

37. During today's visit, did the health worker ask you for the child's Road to Health booklet (RtHB)? 1] Yes 2] No

38. If no, did they ask for the vaccination status of the child? 1] Yes 2] No

39. Was your child vaccinated here today? 1] Yes 2] No

40. If no, why? 1] The health worker said that the child was not eligible for vaccination today. 2] The health workers who saw us did not tell me about vaccinating the child 3] The health worker said that the child could not be vaccinated because s/he was sick 4] The last time the child was vaccinated s/he got sick or had a reaction 5] My religion does not permit vaccination or I don't believe in vaccines 6] Vaccination was not the purpose of this visit 7] The child is already fully vaccinated for his/her age 8] I don't trust the health workers or vaccines in this health facility 9] I forgot to take my child to the vaccination area 10] I didn't have time today to wait for vaccination 11] There were no vaccines in the health facility today 12] There were no syringes or other vaccination supplies 13] Today is not a vaccination day in this health facility 14] The vaccination area was closed 15] The person in charge of vaccination was not there 16] There would have been a long wait 17] The staff treated us badly 18] Other (please specify)

41. If you were advised by a health worker during a clinic visit that your child is missing one or more vaccine doses, and the health worker offers to vaccinate the child with the missed doses, will you refuse? 1] Yes 2] No

**SECTION E: QUALITY OF VACCINATION SERVICES**

**Instruction: Complete this section only if the child received vaccination today**

42. How long did you wait for your child to be vaccinated? _______ Hours _________Minutes
43. Did were you informed what vaccines the child was given? 1] Yes 2] No
44. Today, were you informed the date for the child's next vaccination appointment? 1] Yes 2] No
45. Today, were you given details of the next vaccination appointment? 1] Yes 2] No
46. Did you receive information today on the reactions or side effects that can occur following vaccinations? 1] Yes 2] No
47. If yes, what were you told? 1] Pain at injection sites 2] Fever 3] Rash 4] Diarrhea 5] Vomiting 6] Others (Please specify)
48. Did you receive information today on what you should do if the child has reactions or side effect to the vaccines? 1] Yes 2] No
49. Are you satisfied with the services provided today? 1] Yes 2] No
50. If yes, why? 1] Immediate attention 2] Friendly treatment by staff 3] No charge for services 4] The necessary vaccines and supplies were available 5] Others (Specify)
51. If no, why? 1] Had to wait a long time 2] The staff was discourteous 3] The language that the health worker use is not clear 4] They did not explain what vaccines they had given the child 5] The necessary vaccines and supplies were not available 6] Others (Specify)

**SECTION F: REASONS TO VACCINATE CHILDREN**

52. Could you tell me the purpose of vaccines? (Multiple responses allowed) 1] To prevent diseases 2] So children will grow up healthy 3] To cure diseases 4] They don't do any good 5] Not sure what they are for 6] Other (Please specify)
53. Do you think your child could get these diseases if you don't vaccinate him/her? 1] Yes 2] No 3] Don't know
54. What suggestions do you have to improve vaccination services? 1] There should be more vaccination personnel 2] There should be less waiting time 3] Hours and days when vaccinations are available should not be limited 4] Road to Health

booklet (RtHB) should remain free 5] The treatment of the public and of the children being vaccinated should be friendlier 6] The health center should always have vaccines 7] The should provide information on the vaccines that are being given, on the diseases that they prevent, and on the reactions that they produce. 8] More outreach services 9] None 10] Don’t know 11] Others (Please specify)
